# Combining forces in dual certified centers of geriatric trauma/joint replacement according to the German trauma society (DGU)/endocert improves mobility and quality of life in elderly patients with femoral neck fractures: an analysis from the registry for geriatric trauma (ATR-DGU)

**DOI:** 10.1007/s00068-025-03059-3

**Published:** 2026-01-22

**Authors:** Christopher Lampert, Hannah Schmidt, Yunjie Zhang, Johannes Gleich, Evi Fleischhacker, Carsten Schoeneberg, Bastian Pass, Markus Bormann, Jörg Arnholdt, Boris Michael Holzapfel, Wolfgang Böcker, Maximilian Lerchenberger, Carl Neuerburg, Working Committee on Geriatric Trauma Registry (AK ATR) of the German Trauma Society

**Affiliations:** 1 Department of Orthopaedics and Trauma Surgery, Musculoskeletal University Center Munich (MUM), LMU University Hospital, LMU Munich, Marchioninistr. 15, 81377 Munich, Germany; 2 AUC - Academy for Trauma Surgery, Emil-Riedel-Str. 5, 80538 Munich, Germany; 3 Department of Orthopedic and Emergency Surgery, Alfried Krupp Hospital, Alfried-Krupp-Str. 21, 45131 Essen, Germany; 4 Working Committee on Geriatric Trauma Registry (AK ATR) of the German Trauma Society, Straße des 17. Juni 106-108, Berlin, 10623 Germany; 5 Department of Orthopedics and Traumatology, Paracelsus Medical University, Nuremberg, 90741 Germany

**Keywords:** Hip fracture surgery, Elderly, Geriatric trauma center, Endoprosthesis center, Inpatient mortality, Complications

## Abstract

**Background:**

Femoral neck fractures represent a significant health challenge among elderly. Specialized geriatric trauma centers (ATZ) and endoprosthesis centers (EPZ) have been established to optimize outcomes through interdisciplinary care. This study investigates the impact of combining expertise of ATZ and EPZ on clinical outcomes of elderly patients with femoral neck fractures.

**Methods:**

This retrospective cohort study analyzed data from 25,443 patients aged > 70 years with femoral neck fractures, who were surgically treated in certified ATZ in Germany between 2016 and 2022. Data was obtained from the Registry for Geriatric Trauma (ATR-DGU). Patients were categorized based on treatment at either certified ATZ alone or at combined ATZ with integrated EPZ. Multivariate analyses adjusted for age, gender, ASA score, pre-fracture walking ability, and anticoagulant use were conducted using linear and logistic regression models to assess postoperative outcomes.

**Results:**

Patients treated in dual certified centers had a higher rate of total hip arthroplasty (19.21 vs. 14.18%, *p < *0.001) and received tranexamic acid more frequently (6.20 vs. 1.56%, *p < *0.001). Head-retaining procedures played only a minor role in both centers (8.33% vs. 8.61%, p = 0.452). Despite higher prevalences of severe systemic disease (ASA score > 3), treatment in dual certified centers demonstrated improved early mobilization under full weight-bearing (OR 1.4, 95% CI 1.23–1.6, *p < *0.001) and better mobility at 120 days (OR 1.17, 95% CI 1.03–1.33, p = 0.018).

**Conclusion:**

Treatment in dual certified centers (EPZ/ATZ) is associated with improved postoperative mobility and quality of life in elderly patients with femoral neck fractures. The higher rate of total hip arthroplasty and the combination of geriatric and arthroplasty expertise contribute to these better outcomes.

**Supplementary Information:**

The online version contains supplementary material available at 10.1007/s00068-025-03059-3.

## Introduction

Femoral neck fractures present a major health challenge, particularly among elderly patients, and are associated with high morbidity and mortality rates. These fractures often result in substantial limitations in mobility and quality of life, increasing the need for long-term care and support [1–4]. The treatment of femoral neck fractures in elderly patients requires a specialized and interdisciplinary approach to address both the acute surgical needs and the complexities of geriatric care [5].

Therefore, specialized geriatric trauma centers, that integrate the expertise of trauma surgeons and geriatricians, have become increasingly important in optimizing the treatment and outcome of these fractures [6]. This interdisciplinary approach is associated with improved clinical outcomes as patients regain mobility and independence faster while minimizing postoperative complications such as delirium, and infections [5, 7, 8]. In geriatric trauma centers, the interdisciplinary team typically includes trauma surgeons, geriatricians, physiotherapists, nurses, and social workers. This team collaborates to develop and implement individualized care plans for surgical treatment, pain management, early mobilization, delirium prevention, osteoporosis therapy, and discharge management.. To provide a certain standard of quality these centers are certified by the German Trauma Society (DGU) as AltersTraumaZentrum DGU® (ATZ).

In the treatment of femoral neck fractures, arthroplasty has become established in older patients, particularly due to the immediate full weight bearing [9]. Early mobilization with full weight-bearing plays a major role for the recovery of elderly patients [10]. The choice between total hip arthroplasty (THA) and hemiarthroplasty (HA) remains controversial. HA is considered to be a faster operation with shorter operation times, less blood loss and less surgical stress, which can lead to fewer postoperative complications in this frail patient group [11, 12]. THA has been favored in more active and mobile patients to improve functional outcomes [13]. However, the most recently published prospective randomized trial found similar reoperation rates within the first 2 years but higher dislocation rates in the THA group with only modest clinical improvements [14]. Surgical outcomes especially after arthroplasty are known to be superior in high volume hospitals [15]. Fewer surgical complications like surgical site infections as well as a reduction in mortality rates are reported [16].

In 2012 the EndoCert initiative in Germany was established as the first worldwide certification system of medical centers for arthroplasty [17]. Centers must meet stringent criteria for certification as a specialized endoprosthesis center (EndoProthetikZentrum, EPZ), among other things, at least 100 arthroplasty procedures (including revision operations) must be performed per year. EPZ also follow a multidisciplinary approach, but in contrast to ATZ focus more on the surgical and post-operative aspects of care. Postoperative care in these centers is geared towards rapid rehabilitation and minimizing complications. Particularly in the group of vulnerable patients, a further reduction of surgical risks and an earlier recovery are of great value.

This study aims to investigate the influence of a combined approach of a certified geriatric trauma center with a certified endoprosthesis center on postoperative mobility and quality of life of patients with femoral neck fractures.

## Materials and methods

Patients who suffered a femoral neck fracture and were treated in a hospital certified as ATZ were included. For this study, patients were divided into two groups: firstly, Patients who were treated in a solely certified ATZ (ATZ), and secondly patients who were treated in a hospital that is also a certified EPZ in addition to being certified as an ATZ (EPZ/ATZ). Data sets that have been entered in the Registry for Geriatric Trauma (ATR-DGU) between 2016 and 2022 from 145 German hospitals were retrospectively analyzed. Peritrochanteric, periprosthetic and pathologic femoral fractures were not included in our study.

In Germany, hospitals treating geriatric trauma patients can obtain certification as a Geriatric Trauma Center (AltersTraumaZentrum DGU®, ATZ) by the German Trauma Society (Deutsche Gesellschaft für Unfallchirurgie, DGU). This certification ensures standardized interdisciplinary management of elderly trauma patients, including collaboration between trauma surgeons, geriatricians, physiotherapists, nurses, and social workers according to defined quality criteria. Similarly, hospitals performing hip and knee arthroplasty may be certified as Endoprosthesis Centers (EndoProthetikZentrum, EPZ) within the EndoCert initiative, which establishes minimum procedural volumes, surgical expertise, and structured perioperative protocols for joint replacement surgery. The EndoCert system differentiates between two certification levels (EPZ and EPZmax) according to annual case volume and the scope of revision procedures performed. In our study, 40 EPZ and 40 EPZmax centers were included. For analysis, both certification levels were evaluated together.

The primary outcome of this study was postoperative mobility and quality of life, assessed through early mobilization, walking ability at 7 and 120 days after surgery, and EQ-5D-5L index values. Secondary outcomes included full weight-bearing on day 1, revision surgery, mortality, non-surgical complications, and initiation of osteoporosis treatment.

The ATR-DGU is a multicenter database that collects standardized information on elderly patients undergoing hip fracture surgery. Details of data collection methods, collected information, and registry infrastructure has been described in detail previously and is provided in the Supplementary Materials (Supp. 1) [18, 19]. This study, identified by project number ATR-2022–005, follows the Strengthening the Reporting of Observational Studies in Epidemiology (STROBE) guidelines for cohort studies. The Ethics Committees of the Medical Faculty of LMU Munich, Germany (Reg. No. 234–16) and the Medical Faculty of Philipps-University Marburg, Germany (AZ 46/16) have both approved the data analysis.

For descriptive analyses, categorical data were summarized as counts and percentages, while continuous variables were reported as mean with standard deviation (SD). Due to some patients having missing data for certain parameters, the number of analyzed patients is indicated for each analysis. Results of the EQ-5D-5L questionnaires were transformed to a single index value, using the German (GER) Ludwig value set Version 2.1[20]. For patients with documented EQ-5D-3L questionnaire results, cross walk mapping was performed to obtain the EQ-5D-5L index value. This index value ranges from −0.661 for the worst to 1 for the best health status. Group comparisons were conducted using the 2-sided t-test for continuous variables and the Chi-squared-test for categorical variables. To assess the impact of treatment in dual certified centers for ATZ and EPZ on various outcomes at 7- and 120-days post-surgery, linear and logistic regression models were employed. All multivariate analyses were adjusted for age, gender, and ASA score, pre-existing anticoagulant drugs, and walking ability pre fracture. These variables were selected because they represent key confounders known to influence postoperative outcomes in elderly patients with femoral neck fractures. The ASA score was included as an indicator of comorbidity burden, preoperative anticoagulant use as a risk factor for perioperative complications and revision surgery, and pre-fracture walking ability to better assess the primary outcome of postoperative mobility. Results are expressed as regression coefficients (ß) for linear regression and Odds Ratios (OR) for logistic regression, with 95% confidence intervals (CI). A p-value < 0.05 was considered statistically significant. Significant differences between the groups regarding baseline characteristics, type of surgery and perioperative parameters could be attributed to the large sample size and therefore should be interpreted with caution. Statistical analyses were performed using the R v. 4.0.2 software (Foundation for Statistical Computing, Vienna, Austria).

## Results

**Fig. 1:**
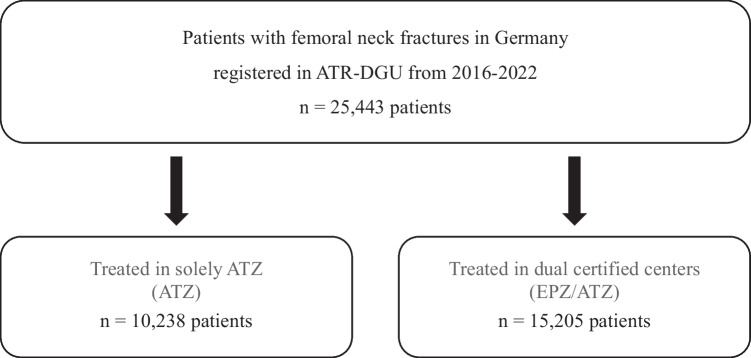
Flow chart presenting inclusion process during study period

A total of 25,443 patients with femoral neck fractures who underwent surgery in 145 ATZ in Germany were included in our study. 15,205 patients were treated in dual certified hospitals according to the German Trauma society/EndoCert (EPZ/ATZ) (Fig. 1).

The mean age of the study population was 83.7 years with more than two third being women in each group (68.71% and 69.36%, respectively). Only 38% of the patients could walk by themselves without walking aids before the fracture occurred. There was no difference in baseline data regarding age, sex and walking ability between the two groups. Interestingly 77.5% of the patients at the EPZ/ATZ and only 71.4% of the patients, who were treated at the solely ATZ certified centers, had severe systemic disease (defined as ASA-Score > 3). Anticoagulant medication was taken by 51.8% of the patients treated at the solely ATZ and 53.9% of the patients at the combined EPZ/ATZ (Table 1).

**Table 1:** Baseline characteristics of patients suffering from femoral neck fractures

	**ATZ**		**EPZ/ATZ**		***P*-value**
Total patients n	10,238		15,205		
Age years (n = 25,170)					
Mean ± SD	83,6 ± 6.47		83.76 ± 6.44		0.052
	n	%	n	%	
Sex (n = 25,400)					
Women	7,026	68.71	10,525	69.36	0.283
ASA-Score (n = 24,974)					** < 0.001**
I	263	1.63	131	0.87	
II	2,698	27.00	3,234	21.58	
III	6,474	64.80	10,408	69.47	
IV	641	6.42	1,201	8.02	
V	15	0.15	9	0.06	
Anticoagulant drugs (n = 24,617)					
Yes	5,117	51.83	7,944	53.88	**0.002**
No	4,756	48.17	6,800	46.12	
Walking ability pre fracture (n = 23,474)					0.069
Without aids	3,618	38.53	5,362	38.07	
With 1 crutch/cane	1,050	11.18	1,473	10.46	
With 2 crutches/walker	2,907	30.96	4,576	32.49	
Only at home	1,530	16.29	2,284	16.22	
None	285	3.04	389	2.76	
Fracture classification (n = 24,101)					0.142
Garden 1 and 2	2,402	24.74	3,683	25.59	
Garden 3 and 4	7,306	75.26	10,710	74.41	

Significant p values are in bold font

Mean value comparisons are carried out using a 2-sided *t*-test, *Chi*-squared test was used for categorical variables

ASA American Society of Anesthesiologists

**Fig. 2:**
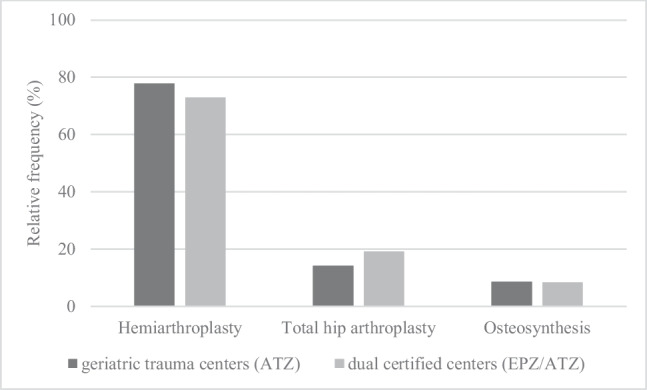
Impact on the type of surgical treatment of femoral neck fractures by center type

Patients who are treated for a femoral neck fracture in a geriatric trauma center with an integrated EPZ received a total hip arthroplasty more frequently (19.21 vs. 14.18%, *p < *0,001), while hemiarthroplasties were implanted correspondingly less frequently (72.95 vs. 77.83%, *p < *0,001). Head-retaining procedures such as osteosynthesis played only a minor role in both groups (8.33% ATZ vs. 8.61% EPZ/ATZ, p = 0.452), indicating no significant difference between center types (Fig. 2).

Tranexamic acid was used more frequently in EPZ/ATZ centers (6.20 vs. 1.56%, *p < *0.001). The most common form of anesthesia at both centers was general anesthesia (92.70% EPZ/ATZ, 90.10% ATZ). Spinal anesthesia was performed in less than 10% of the cases. Postoperative treatment in the intensive care unit was rarely necessary at EPZ/ATZ centers (25.78 vs. 31.28%, *p < *0.001) despite the sicker patients. Compared to the solely ATZ certified centers, there were more unplanned admissions to ICU due to intraoperative complications (12.82 vs. 9.05%, p = 0.026). About one third of the patients were in the intensive care unit for more than 24 h (Table 2).

**Table 2:** Perioperative parameters after operative treatment of femoral neck fractures in ATZ and EPZ/ATZ

	**ATZ**		**EPZ/ATZ**		***P*-value**
	**n**	**%**	**n**	**%**	
Tranexamic acid*	35	1.56	175	6.20	** < 0.001**
Form of anesthesia					
General anaesthesia	9,229	90.10	13,941	92,70	
Spinal anaesthesia	875	8.50	1,110	7,30	
Postoperative ICU*	742	31.28	780	25.78	** < 0.001**
Planned	653	90.95	653	87.18	**0.026**
Intraop complications	65	9.05	96	12.82	
> 24 h	221	30.40	238	31.19	0.783
< 24 h	506	69.60	525	68.81	

* levied from 2022

Significant p values are in bold

*Chi*-squared test was used for categorical variables

ICU Intensive care unit

Patients treated at solely ATZ certified centers suffered less frequent non-surgical complications after hip fracture surgery compared to patients at EPZ/ATZ (33.62 vs 38.34%, *p < *0.001). Complications were comparable between the two center types. Interestingly postoperative delirium was the most common complication at both centers in 27.6% at ATZ and 26.4% at EPZ/ATZ of the cases, followed by urinary tract infections, pneumonia, and acute kidney failure. The documented complications are listed and sorted by frequency in Table 3.

**Table 3:** Non-surgical complications during hospital stay

	**ATZ**		**EPZ/ATZ**	
	**n**	**%**	**n**	**%**
At least one non-surgical complication during hospital stay*	780	33.62	1,142	38.34
Delirium	215	27.6	302	26.4
Urinary tract infection	164	21.0	267	23.4
Pneumonia	131	16.8	179	15.7
Acute kidney failure	116	14.9	196	17.2
Decubitus	64	8.2	55	4.8
Pulmonary embolism	23	2.9	33	2.9
Myocardial infarction	12	1.5	25	2.2
Deep venous thrombosis	6	0.8	15	1.3
Other	344	44.1	504	44.1

* levied from 2022

Treatment in a geriatric trauma center with a specialized EPZ showed a significant impact on mobilization on the first day, full weight bearing from the beginning and initiation of an osteoporosis treatment during in-hospital stay. Although mobility was impaired seven days after surgery, increased mobility was observed in patients treated at EPZ/ATZ after 120 days. Regarding the postoperative outcome non-surgical complications were more likely if the operation was performed at a geriatric trauma center with integrated EPZ. Odds for inpatient mortality and revision operations showed no difference between the two center types (Table 4). Patients treated at solely ATZ certified centers died in 5.81% of the cases compared to 5.72% of the patients treated at a combined center with EPZ/ATZ (p = 0.778). The revision rate was 3.85% at ATZ and 4.02% at EPZ/ATZ, respectively (p = 0.542).

**Table 4:** Multivariable logistic and linear regression analysis of the impact of treatment at EPZ/ATZ on various outcomes during hospital stay

	**n**	**OR**	**95%-CI**	***P*-value**
Mobilization carried out on day 1 after surgery ^**a**^ Full weight bearing on day 1 permitted ^**a**^	21,878 22,025	1.14 1.40	1.17–1.34 1.23–1.60	** < 0.001** ** < 0.001**
Walking ability 7 days after surgery (independent or with walking aid) ^**a**^	21,292	0.92	0.85–1.00	**0.039**
Revision operation performed ^**a**^	20,849	1.02	0.88–1.17	0.796
Non-surgical complications ^**a**^	4,494	1.15	1.02–1.31	**0.026**
Inpatient mortality ^**a**^	20,973	0.90	0.80–1.03	0.119
Osteoporosis treatment 7 days after surgery ^**a**^	13,840	1.15	1.07–1.23	** < 0.001**
	**n**	**β**	**95%-CI**	***P*-value**
EQ-5D-5L index 7 days after surgery ^b^	9,866	0.03	0.02–0.04	** < 0.001**

Reference: Treatment at solely specialized geriatric trauma centers (ATZ), significant p values are in bold, n numbers of observation. All models were adjusted for sex, age, American Society of Anesthesiologists (ASA)-Score, pre-existing anticoagulant drugs and walking ability pre fracture

^a^ Linear regression analysis

^b^ Logistic regression analysis

Logistic regression analysis showed no impact of treatment at EPZ/ATZ on revision operations, mortality and osteoporosis treatment in the medium term follow-up after 120 days. Treatment at EPZ/ATZ showed a positive influence on the quality of life after 7 days. This was also persistent after 120 days (Table 5).

**Table 5:** Multivariable logistic and linear regression analysis of the impact of treatment at EPZ/ATZ on various outcomes after 120 days of follow-up

	**n**	**OR**	**95%-CI**	***P*-value**
Walking ability 120 days after surgery (independent or with walking aid) ^**a**^	6,500	1.17	1.03–1.33	**0.018**
Revision operation performed ^**a**^	7,470	0.97	0.76–1.25	0.816
Death ^**a**^	6,999	0.87	0.72–1.04	0.120
Osteoporosis treatment 120 days after surgery ^**a**^	5,078	0.89	0.78–1.00	0.058
	**n**	**β**	**95%-CI**	***P*-value**
EQ-5D-5L index 120 days after surgery ^b^	2,522	0.08	0.04–0.12	** < 0.001**

Reference: Treatment at solely specialized geriatric trauma centers (ATZ), significant p values are in bold, n numbers of observation. All models were adjusted for sex, age, American Society of Anesthesiologists (ASA)-Score, pre-existing anticoagulant drugs and walking ability pre fracture

^a^ Linear regression analysis

^b^ Logistic regression analysis

## Discussion

Femoral neck fractures in the elderly represent a substantial burden, leading to prolonged disability and high mortality rates. Interdisciplinary treatment approaches can reduce mortality and improve functional outcomes in elderly patients with hip fractures by addressing both surgical and geriatric needs [5]. Previous literature demonstrated that increased geriatric involvement enhances early mobilization, mobility and initiation of osteoporosis treatment [18]. To evaluate the influence of combination of the expertise from specialized geriatric trauma centers and endoprosthesis centers according to the German Trauma society (DGU)/EndoCert on the treatment of femoral neck fractures an analysis of the Geriatric Trauma Registry (ATR-DGU) was conducted. All hospitals that have entered data in the registry are certified ATZ. Therefore, a standardized interdisciplinary orthogeriatric care can be assumed [21, 22].

One of the key findings of our study, is that treatment at combined centers for EPZ and ATZ has positive impact on early mobilization and immediate full weight-bearing. This is crucial in reducing the long-term dependency often associated with femoral neck fractures [10]. Early mobilization is critical for preventing the cascade of complications associated with immobility, such as pneumonia, deep vein thrombosis, and pressure ulcers. It is known that weight-bearing restrictions cannot be maintained by older people and therefore reduce mobility and postoperative outcome of these patients [23]. The improvement in patients' quality of life as early as seven days post-surgery and sustained up to 120 days underline these findings and indicate that the benefits of combined care extend beyond immediate postoperative recovery.

Notably, patients treated in combined centers for EPZ/ATZ were more likely to receive THA rather than HA supports existing literature. High-volume centers often have more experienced surgeons and due to the confidence in handling they tend to THA as it may offer better long-term functional outcomes in selected patients[24]. Particularly patients who are expected to have longer survival and higher functional demands seem to benefit [25, 26]. However, the literature is divided on this point, with some studies indicating that HA, being less invasive and quicker, is preferable for frail patients due to the reduced surgical burden [27, 28]. Among independently ambulating patients with displaced femoral neck fractures, who were randomly assigned to undergo total hip arthroplasty or hemiarthroplasty, serious adverse events, hip-related complications and dislocations were more frequent with total hip arthroplasty [14]. On the other hand, a meta-analysis reported significantly lower risk for reoperation after total hip arthroplasty compared to hemiarthroplasty. Indisputable seems to be an increased risk of dislocation in THA [29]. Head-retaining treatment plays a minor role in elderly patients with femoral neck fractures. In our study, only 8.33% of patients treated in ATZ and 8.61% treated in EPZ/ATZ underwent internal fixation with joint-preserving. These findings reflect the global consensus that arthroplasty should be considered the standard of care for elderly patients with displaced femoral neck fractures [30]. For example, the NICE Guideline (UK, 2023) and the American Academy of Orthopaedic Surgeons (AAOS, 2022) provide comparable recommendations, stating that in elderly patients with displaced femoral neck fractures, primary arthroplasty is recommended to promote early mobilization under full weight bearing postoperatively [31, 32]. Intraoperative use of tranexamic acid (TXA) is widespread in arthroplasty, as it is known to reduce the administration of allogenic blood transfusion [33]. In line with this, patients treated at in a geriatric trauma center with an integrated EPZ, whose focus is on standardization and optimization of perioperative structures, TXA was used more frequently. The reduction of intraoperative blood loss and consecutively cutting the number of blood transfusions is crucial to improve the postoperative outcome in elderly after hip fracture surgery [34].

Interestingly, patients who were treated in the EPZ/ATZ had more severe pre-existing conditions and were more likely to be taking anticoagulant medication. Patients with significant comorbidities are often referred to specialized centers, particularly those that are high-volume, due to the need for advanced surgical care and multidisciplinary teams to manage perioperative risks. Despite the fact of the higher baseline severity of illness in patients treated at dual certified centers for EPZ/ATZ, as indicated by a greater proportion of patients with an ASA score > 3, fewer patients were admitted to ICU postoperatively. In particular, the lower proportion of planned admission to ICU for routine monitoring emphasizes the handling of complex cases at specialized high-volume centers.

The rate of revision operations and mortality was not different between patients treated at solely ATZ certified centers or those with integrated EPZ. However, our data also suggest that non-surgical complications were more frequent when treated in dual certified EPZ/ATZ centers. The inpatient mortality rate (5.81% ATZ and 5.72% EPZ/ATZ) was comparable to existing literature with reported rates between 2.9—5.4% [35, 36]. Fittingly to our results association between hospital volume and outcome varied [37]. Particularly postoperative delirium, remains a significant concern, occurring at similar rates in both types of centers. Patients who experienced delirium have an increased risk of worse outcomes as it correlates with a longer hospital stay, as well as an increased association with complications, and higher mortality [38, 39]. This highlights the need for ongoing efforts to improve delirium prevention strategies through enhanced geriatric co-management. Furthermore, our results illustrate that treatment of an underlying osteoporosis is influenced by a treatment at EPZ/ATZ. This is particularly important because hip fracture patients face twice the risk of experiencing a secondary fracture, and adherence to osteoporosis therapy reduces the risk of subsequent fractures and mortality [40, 41].

This study has several limitations that must be considered. The retrospective design, while allowing for the inclusion of a large number of patients, is inherently subject to selection bias. The study's findings may not be generalizable to all healthcare settings, as it was conducted in certified ATZ in Germany. Additionally, the heterogeneity of the patient population, with varying degrees of comorbidities, could introduce variability in the outcomes. Despite adjusting for several confounding factors, unmeasured variables may also affect the results. There was no information whether patients had pre-existing hip arthritis or arthritic pain prior to the injury. These factors can substantially influence the choice of surgical treatment and should be considered potential confounders. Moreover, the study relies on the accuracy and completeness of data entered in the registry, which, although standardized, may still vary across participating centers. Data on the use of tranexamic acid, postoperative intensive care therapy and non-surgical complications must be interpreted with caution due to the short data collection period and the associated comparatively low number of patients. The short follow-up period may not capture long-term outcomes, and variability in surgical techniques and care protocols across centers could further influence the findings. Lastly, potential selection bias in the choice between total hip arthroplasty and hemiarthroplasty may impact the comparison of these treatment options.

## Conclusion

This study demonstrates that treatment in dual certified centers for geriatric trauma and arthroplasty care (EPZ/ATZ) is associated with improved postoperative mobility and quality of life in elderly patients with femoral neck fractures. The higher rate of total hip arthroplasty observed in dual certified centers may contribute to this benefit, as THA improve functional recovery in selected patients. Nevertheless, our results suggest that the combination of geriatric and arthroplasty expertise, with interdisciplinary management and optimized perioperative care, plays a key role in achieving better functional outcomes. These findings highlight the potential of integrated care structures as a model for future treatment strategies in geriatric fracture management.

## Supplementary Information

Below is the link to the electronic supplementary material.

Supplementary file1 (DOCX 15 KB)

## Data Availability

The data that support the findings of this study are derived from the AltersTraumaRegister DGU® (ATR-DGU) of the German Trauma Society (Deutsche Gesellschaft für Unfallchirurgie, DGU). Data access is subject to approval by the AltersTraumaRegister DGU® committee and cannot be made publicly available due to data protection regulations. Researchers may request access to the data directly from the AltersTraumaRegister DGU® of the DGU.
