# Supplementary material for: Combining forces in dual certified centers of geriatric trauma/joint replacement according to the German trauma society (DGU)/endocert improves mobility and quality of life in elderly patients with femoral neck fractures: an analysis from the registry for geriatric trauma (ATR-DGU)
Source: Eur J Trauma Emerg Surg. 2026 Jan 22;52(1):26. doi: 10.1007/s00068-025-03059-3 (PMC12827371; doi:10.1007/s00068-025-03059-3)
Supplement: Supplementary file 1 — Supplementary file1 (DOCX 15 KB) [file 68_2025_3059_MOESM1_ESM.docx]

**Supplementary 1**

The ATR-DGU is a multicenter database established by the German Trauma Society (DGU) in 2016. Hospitals certified as ATZ are required to enter detailed information about their patients aged 70 years and older who suffer from hip fractures and underwent surgery. Data is collected using standardized questionnaires and comprises five consecutive time phases: Admission, preoperative phase, operation, post-operative phase, discharge and an optional follow-up examination on day 120 after the operation. The collected data encompasses patient details (age and sex), walking ability before the incident, existing level of care, anticoagulant and osteoporosis medication use at admission, and a geriatric assessment. Concerning the surgical part, information on fracture configuration, surgical and anesthesia procedures, and ASA-Classification (American Society of Anesthesiologists) is recorded. In the postoperative phase, data on walking ability, initiation of osteoporosis treatment, revision operations, and non-surgical complications are documented. Discharge data include the patient's destination as well as inpatient mortality rate. Follow-up assessments 120 days post-surgery were carried out including questions about walking ability, revision operations, as well as health-related quality of life questionnaires. Data on the use of tranexamic acid, postoperative intensive care therapy and non-surgical complications have only been recorded in the ATR-DGU since 2022.

The AUC—Academy for Trauma Surgery (AUC), affiliated with the German Trauma Society (DGU), provides and maintains the infrastructure for data entry, management, and analysis. Scientific leadership is the responsibility of the Working Committee on Geriatric Trauma Registry (AK ATR) of the DGU. Approval for scientific data analysis from the Registry for Geriatric Trauma (ATR-DGU) is granted through a peer-review process in accordance with publication guidelines established by the AK ATR of the DGU.
